# Impact of femoral head size on the localization of proximal femur fractures: Retrospective analysis of 400 cases

**DOI:** 10.1016/j.jcot.2025.103007

**Published:** 2025-04-02

**Authors:** Nele Wagener, Gregor Giebel, Felix Rarreck, Torsten Diekhoff, Sebastian Hardt

**Affiliations:** a Charité Universitätsmedizin Berlin, Corporate Member of Freie Universität Berlin and Humboldt-Universität zu Berlin, Center for Musculoskeletal Surgery, Department of Orthopaedic Surgery, Charitéplatz 1, 10117, Berlin, Germany; b Department of Radiology, Charité-Universitätsmedizin Berlin, Campus Mitte, Humboldt-Universität zu Berlin, Freie Universität Berlin, Berlin, Germany

**Keywords:** Hip fractures, Femur head, Fractures, Bone, Osteoarthritis, Hip, Radiography

## Abstract

**Background:**

Proximal femur fractures are prevalent among the elderly, leading to high mortality, reduced quality of life, and significant healthcare burdens. The incidence is rising with demographic ageing, with osteoporotic fractures projected to reach 6 million annually by 2050, costing $25.4 billion. A 351 % increase in proximal femur fractures among individuals over 85 years is expected. Accurate fracture localization through imaging, combined with understanding femoral head size and patient-specific factors, improves preoperative planning and outcomes. This study explores the relationship between femoral head size and fracture localization.

**Methods:**

A retrospective cohort study analyzed data from 400 patients with proximal femur fractures treated between 2010 and 2022. Fractures were classified as medial, lateral, pertrochanteric, or subtrochanteric. Radiographs measured femoral head morphology, and statistical analyses, including chi-square tests, t-tests, ANOVA, and logistic regression, identified predictors of fracture localization.

**Results:**

Femoral head area (FHA) varied significantly across fracture types, with lateral fractures having the largest mean FHA (2355.95 mm^2^/cm^2^, *p* = 0.047). Osteoarthritis prevalence differed (*p* = 0.028), being highest in subtrochanteric fractures (17 %, Kellgren & Lawrence grade 3–4). Lateral fractures had a younger mean age of 71.05 years (*p* < 0.001), while pertrochanteric fractures averaged 79.52 years (*p* < 0.001). Vertical (*p* < 0.001) and horizontal (*p* = 0.028) femoral head diameters also differed significantly.

**Conclusion:**

Larger femoral heads are associated with lateral fractures, whereas pertrochanteric fractures occur in older patients. Subtrochanteric fractures correlate with smaller femoral heads and advanced osteoarthritis.

## Abbreviations

**AI**: - Acetabular Index
**ANOVA**: - Analysis of Variance
**AP**: - Anteroposterior
**BH**: - Body Height
**BMI**: - Body Mass Index
**CCD**: - Centrum-Collum-Diaphyseal Angle
**CT**: - Computed Tomography
**FHA**: - Femoral Head Area
**FHD**: - Femoral Head Diameter
**FNA**: - Femoral Neck Axis Length
**FNAL**: - Femoral Neck Axis Length
**FND**: - Femoral Neck Diameter
**FO**: - Femoral Offset
**HAL**: - Hip Axis Length
**HRV**: - Head Radius Vertical
**HRV/FNA**: - Head Radius Vertical/Femoral Neck Axis
**IBM**: - International Business Machines
**MERLIN**: - Measurement Program
**NSA**: - Neck Shaft Angle
**NW**: - Neck Width
**PACS**: - Picture Archiving and Communication System
**PFNA**: - Proximal Femoral Nail Advanced
**ROI**: - Region of Interest
**SD**: - Standard Deviation
**SPSS**: - Statistical Package for the Social Sciences
**THA**: - Total Hip Arthroplasty

## 1 Introduction

Proximal femur fractures are among the most common and serious injuries in older patients, associated with high mortality rates and significant reductions in quality of life.^1^, ^2^, ^3^ The incidence of these fractures increases with age, posing a significant burden on healthcare systems. Additionally, patient age is a known risk factor for fracture development, with older patients being more susceptible due to osteoporosis and other degenerative skeletal changes.^2^^,^^4^, ^5^, ^6^

In Western countries, the incidence is approximately 100–200 cases per 100,000 individuals per year, rising to up to 500 cases per 100,000 individuals per year in those over 80 years old.^7^ Women are more frequently affected than men, with about twice the incidence in women, primarily due to the higher prevalence of osteoporosis.^8^^,^^9^

With the demographic aging of the population, the number of proximal femur fractures worldwide is expected to rise to about 6 million per year by 2050, leading to considerable socioeconomic challenges.^10^^,^^11^

The treatment of proximal femur fractures depends on the type of fracture, the patient's age, bone quality, and functional requirements. In patients with poor bone quality and complex fractures, surgical treatment is often preferred. The most common surgical procedures include hip arthroplasty (total or partial) and internal fixation with screws, nails, or plates.^12^, ^13^, ^14^ In modern traumatology, conservative treatment of such injuries plays a minor role. Postoperative care focuses on early mobilization, pain management, and rehabilitation to promote functional recovery and minimize complications. It is important to emphasize that young patients are often treated with joint-preserving surgery, whereas in older patients, there is a tendency towards total or partial hip replacement. Additionally, pertrochanteric fractures are typically managed with intramedullary nailing.^15^, ^16^, ^17^

Imaging techniques are crucial for the diagnosis and classification of fractures. Standard practice includes taking radiographs in two planes, with CT scans being used in more complex cases or when radiographic findings are unclear, providing additional information on fracture morphology and the extent of injury.^18^^,^^19^ Several biomechanical factors may influence the relationship between femoral head size and fracture patterns. For instance, a larger femoral head may alter the load transmission and increase the lever arm acting on the proximal femur, potentially leading to different stress distributions and fracture types.^20^ Conversely, a smaller or degeneratively altered femoral head may be less effective in dissipating mechanical loads, contributing to fracture occurrence in other anatomical regions.^21^^,^^22^ Variations in femoral geometry—such as neck-shaft angle, offset, or femoral neck length—could further affect mechanical loading and susceptibility to specific fracture patterns.^23^^,^^24^

Moreover, ethnical and geographical differences in femoral head size and morphology have been documented. Studies suggest that populations of Asian or African descent may have distinct proximal femur geometries compared to Caucasian populations, which can influence fracture incidence and localization.^24^, ^25^, ^26^, ^27^ These anthropometric variations should be considered when analyzing fracture mechanisms and developing treatment strategies.

The aim of this study is to explore the potential biomechanical factors that influence fracture localization. We hypothesize that the size of the femoral head could be indirectly associated with specific fracture locations due to its correlation with anatomical landmarks or fracture patterns. Variations in femoral head size might reflect underlying biomechanical properties of the bone that predispose certain areas to fracture under load. Understanding these relationships could provide supplementary information when standard imaging is inconclusive, ultimately enhancing preoperative planning and improving surgical interventions and outcomes for patients. So far, no studies have thoroughly examined the relationship between anatomical differences and type of fracture.

Therefore, the specific research question of this study was: Does the size and shape of the femoral head influence the localization of proximal femur fractures, and how are these morphological parameters associated with patient-related factors such as age and osteoarthritis?

## 2 Methods

The study was approved by the hospital's ethics committee (EA1/203/23) and informed consent was waived due to the retrospective nature of the study and follows the ethical principles of the Helsinki Declaration.

**Fig. 1:**
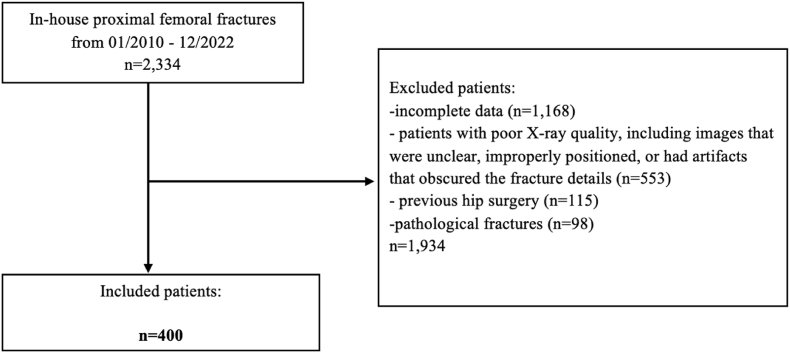
Flow-chart of patient selection.

This monocentric, retrospective cohort study analyzed data from patients diagnosed with proximal femur fractures (medial, lateral, pertrochanteric, subtrochanteric) at a major academic medical center between January 2010 and December 2022. Initially, a total of 2334 patients with proximal femoral fractures were identified (Fig. 1). Patients were included and separated into groups based on fracture localization, with 100 consecutive patients selected for each group to ensure balanced representation. Excluded were patients with incomplete data, poor X-ray quality, previous hip surgery, and pathological fractures. In total, 400 patients aged 18–100 years were included in our study.

Fracture classification was based on standardized radiological criteria.

Medial and lateral femoral neck fractures were defined according to their location relative to the mid-cervical axis: Medial femoral neck fractures included subcapital and transcervical fractures, located proximal to the mid-cervical line. Lateral femoral neck fractures were defined as basicervical or fractures involving the lateral third of the femoral neck. Pertrochanteric fractures were defined as those involving the region between the greater and lesser trochanters, while subtrochanteric fractures were defined as those located up to 5 cm distal to the lesser trochanter.

Osteoarthritis was graded using the Kellgren & Lawrence classification (grades 0–4) based on the presence of joint space narrowing, osteophyte formation, subchondral sclerosis, and deformity of the femoral head. This classification was assessed using preoperative pelvic radiographs by three independent raters, with disagreements resolved by consensus.

Pelvic radiographs were routinely obtained after trauma with a Direct View system (Brainlab AG, Munich) at our hospital. Images were acquired in standardized anteroposterior (AP) view from approximately 100 cm above the supine patient, with both hips internally rotated by 15° to ensure consistent positioning. The symphysis pubis was centered, and the obturator foramen index served as the reference parameter. To adjust for magnification, a standardized calibration marker (metallic sphere, 25 mm in diameter) was placed at the level of the greater trochanter, enabling accurate measurement of femoral head and neck dimensions by correcting for radiographic distortion.

Based on patient data and preoperative radiographs from electronic patient records, femoral head size and shape were measured for medial, lateral, pertrochanteric, and subtrochanteric femur fractures to identify morphological differences. The determination of radiological parameters of the FHA was carried out using only preoperative pelvic overview radiographs and axial radiographs. In axial radiographs, the femoral head contour was manually traced in the axial plane at the level of the maximal transverse diameter, using a polygonal region of interest (ROI). This allowed for complementary assessment of the femoral head area, particularly in cases where the AP view was limited by projectional distortion. Demographic data collected included age, sex, and the presence of comorbidities such as cardiovascular, respiratory, digestive, neurological, endocrine, musculoskeletal, psychiatric, renal, infectious, oncological diseases, osteoporosis, and smoking status.

**Measurement methodology**: All measurements were digitally conducted using the measurement program MERLIN (Diagnostic Workcenter, Phönix-PACS GmbH, Freiburg, Deutschland) on the picture archiving and communication system (PACS). These measurements were independently performed by three orthopedic surgeons and averaged. The following parameters were measured and are defined as follows:

• **FHA (Femoral Head Area)**: Calculated by manually outlining the femoral head contour on the fracture side using a polygonal region of interest (ROI) in the PACS system (mm^2^/cm^2^). Alternative methods such as ellipse-based estimation (A = π × a × b) were considered but not used due to lower accuracy in irregular or osteoarthritic heads.
• **FHD (Femoral Head Diameter)**: Measured in horizontal (mediolateral) and vertical (superoinferior) planes at the widest extent of the femoral head (fracture side).
• **FNA (Femoral Neck Axis Length)**: Distance from the center of a best-fit circle of the femoral head to the base of the femoral neck.
• **HRV (Head Radius Vertical)**: Vertical distance from the superior cortical margin to the center of the femoral head.
• **CCD (Centrum-Collum-Diaphyseal Angle)**: Measured in degrees on the healthy side by drawing axes along the femoral shaft and neck.
• **HRV/FNA Ratio**: Index for femoral head shape assessment, calculated using healthy side data.

**Fig. 2:**
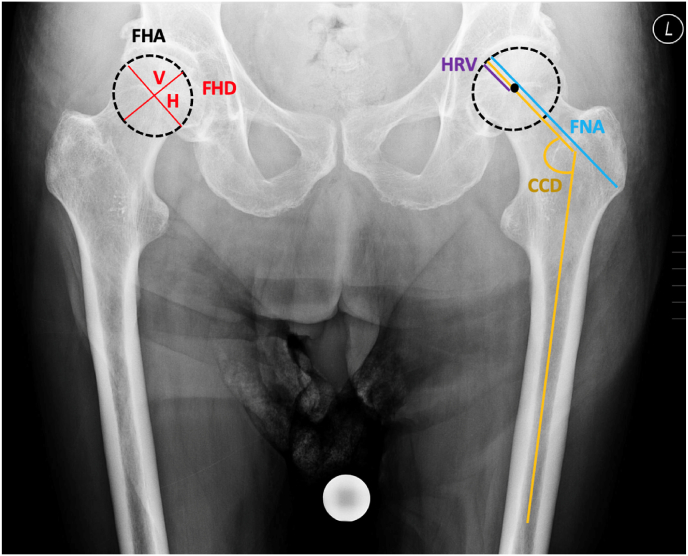
Measurements of femoral head morphology: FHA = femoral head area (mm^2^/cm^2^); FHD = femoral head diameter (mm), V = vertical, H = horizontal (mm); FNA = femoral neck axis length (mm); HRV = head radius vertical (mm); CCD = CCD angle (°); HRV/FNA = head radius vertical divided by femoral neck axis length.

These parameters are visually illustrated and defined in Fig. 2 of the manuscript.

Categorical data are summarized by absolute frequencies (n) and relative frequencies (%), metric variables by mean (M) and standard deviation (SD). First, the data were tested for normal distribution using a Shapiro-Wilk test as well as exploratory by skewness, kurtosis, and histograms. Chi-Square Test or Fisher Exact Test, in case of expected cell frequencies <5, were applied for comparison of categorical variables between the fracture localization groups. For continuous outcomes, comparison between the 4 fracture localization groups was performed using one-way analysis of variance (ANOVA). In case of significant group differences, bivariate post-hoc tests using Bonferroni adjustment to avoid inflation of the family-wise error rate were performed. Effect size (η^2^) shows the relevance of the observed effect. The following rules of thumb are used to interpret values for Eta squared: 0.01 indicates a small effect size, 0.06 a medium effect size, and 0.14 or higher a large effect size 28. Spearman correlation coefficients with 95 % confidence intervals were calculated to analyze the relationship of metric variables. p-values less than 0.05 are considered statistically significant. Calculations were performed using SPSS version 29 (IBM Inc.).

## 3 Results

**Fig. 3:**
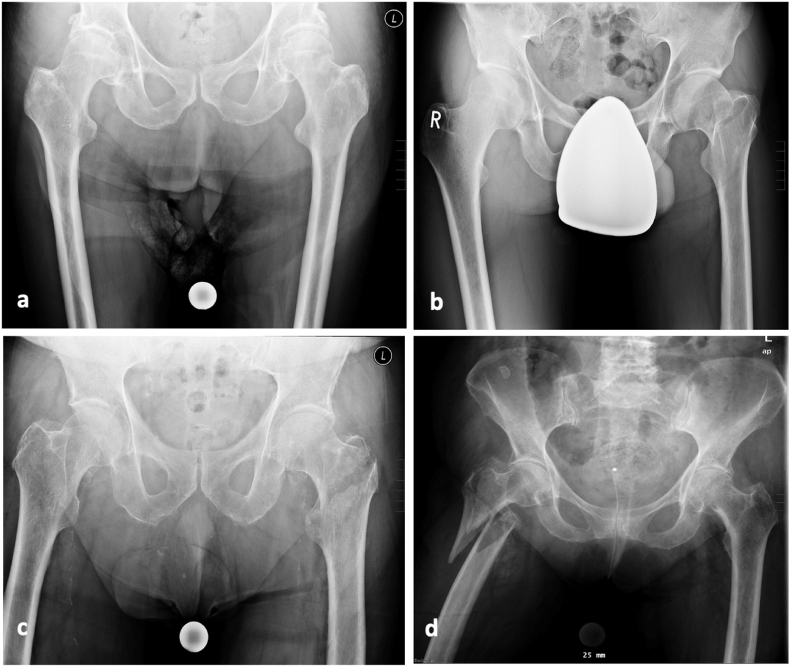
Radiographic images illustrating different types of proximal femur fractures: **(a)** Left medial femoral neck fracture (the right side is healthy); **(b)** Left lateral femoral neck fracture (the right side is healthy); **(c)** Left pertrochanteric femoral fracture (the right side is healthy); **(d)** Right subtrochanteric femoral fracture (the left side is healthy).

We included 100 consecutive patients in each group, resulting in a total of 400 patients diagnosed with proximal femur fractures (medial, lateral, pertrochanteric, and subtrochanteric) at a major academic medical center from 2010 to 2022 (Fig. 3). The study cohort consisted of 225 females (56.25 %) and 175 males (43.75 %), with an average age of 74.44 years (SD = 12.34) at the time of fracture occurrence. All patients underwent surgical treatment. Demographic characteristics and clinical profiles are presented in Table 1.

**Table 1:** **Distribution of health conditions across different types of proximal femur fractures** (Medial, Lateral, Pertrochanteric, and Subtrochanteric). Abbreviations include N (Number of cases), % (Percentage of cases) and Total Hip Arthroplasty (THA). The table shows the presence (Yes) or absence (No) of various health conditions and their significance using chi-square tests (*p*-values <0.05 indicate significant associations). This analysis provides insights into the correlation between health conditions and specific femur fracture types.

		Type of proximal femur fracture				
		Medial	Lateral	Pertrochanteric	Subtrochanteric	
		n = 100	n = 100	n = 100	n = 100	*p-*value
**Localization**	l	46 (46.00 %)	39 (39.00 %)	53 (53.00 %)	51 (51.00 %)	0.179
	r	54 (54.00 %)	61 (61.00 %)	46 (46.00 %)	49 (49.00 %)	
**Sex**	m	44 (44.00 %)	50 (50.00 %)	38 (38.00 %)	43 (43.00 %)	0.419
	f	56 (56.00 %)	50 (50.00 %)	62 (62.00 %)	57 (57.00 %)	
**Osteoarthritis (Kellgren & Lawrence)**	0	19 (19.00 %)	14 (14.00 %)	5 (5.00 %)	7 (7.00 %)	0.028
	1	27 (27.00 %)	25 (25.00 %)	44 (44.00 %)	30 (30.00 %)	
	2	44 (44.00 %)	51 (51.00 %)	41 (41.00 %)	46 (46.00 %)	
	3	9 (9.00 %)	9 (9.00 %)	8 (8.00 %)	15 (15.00 %)	
	4	1 (1.00 %)	1 (1.00 %)	2 (2.00 %)	2 (2.00 %)	
**Cardiovascular disease**	Yes/No	58/42 (58.00 %)	45/55 (45.00 %)	51/49 (51.00 %)	47/53 (47.00 %)	0.274
**Respiratory disease**	Yes/No	16/84 (16.00 %)	23/77 (23.00 %)	15/85 (15.00 %)	10/90 (10.00 %)	0.101
**Digestive disease**	Yes/No	3/97 (3.00 %)	9/91 (9.00 %)	6/94 (6.00)	7/93 (7.00 %)	0.402
**Neurological disease**	Yes/No	22/78 (22.00 %)	15/85 (15.00 %)	23/77 (23.00 %)	15/85 (15.00 %)	0.304
**Endocrine disorder**	Yes/No	29/71 (29.00 %)	17/83 (17.00 %)	20/80 (20.00 %)	23/77 (23.00 %)	0.214
**Musculoskeletal disorder**	Yes/No	21/79 (21.00 %)	28/72 (28.00 %)	20/80 (20.00 %)	17/83 (17.00 %)	0.293
**Psychiatric disorders**	Yes/No	5/95 (5.00 %)	6/94 (6.00 %)	4/96 (4.00 %)	7/93 (7.00 %)	0.882
**Kidney diseases**	Yes/No	16/84 (16.00 %)	24/76 (24.00 %)	23/77 (23.00 %)	13/87 (13.00 %)	0.143
**Infectious diseases**	Yes/No	11/89 (11.00 %)	5/95 (5.00 %)	7/93 (7.00 %)	2/98 (2.00 %)	0.071
**Oncological diseases**	Yes/No	6/94 (6.00 %)	10/90 (10.00 %)	8/92 (8.00 %)	4/96 (4.00 %)	0.402
**Osteoporosis**	Yes/No	10/90 (10.00 %)	13/87 (13.00 %)	9/91 (9.00 %)	9/91 (9.00 %)	0.780
**Smoking**	Yes/No	25/75 (25.00 %)	23/76 (23.23 %)	21/79 (21.00 %)	13/87 (13.00 %)	0.161

### 3.1 Impact of femoral head size and health conditions on fracture localization

The FHA was significantly different among fracture types, with lateral fractures having a mean area of 2355.95 ± 2110.35 mm^2^, medial fractures 2018.71 ± 394.24 mm^2^, subtrochanteric fractures 1995.72 ± 409.26 mm^2^, and pertrochanteric fractures 1972.96 ± 340.14 mm^2^ (*p* = 0.047) (Table 4). The prevalence of osteoarthritis varied significantly among fracture types (*p* = 0.028), with the highest incidence in lateral fractures (Table 1). However, no significant differences were observed in the prevalence of cardiovascular, respiratory, digestive, neurological, endocrine, musculoskeletal, psychiatric, kidney, infectious, and oncological diseases, as well as osteoporosis and smoking, across fracture types (all *p* > 0.05). The distribution of fractures did not significantly differ by sex (*p* = 0.419) or by fracture localization (*p* = 0.179). Furthermore, the presence of arthroplasty on the healthy side did not significantly differ among fracture types (*p* > 0.999).

### 3.2 Age and BMI analysis of proximal femur fracture types

For age, significant differences were found between the fracture types (Table 2). The overall *p*-value for age across the different fracture types is less than 0.001, indicating a significant difference. Specifically, the pairwise comparisons show significant differences between lateral and pertrochanteric fractures (*p* < 0.001) and between pertrochanteric and subtrochanteric fractures (*p* = 0.002). In contrast, for BMI, no significant differences were observed between the fracture types, as indicated by a *p*-value of 0.065. In summary, there are significant differences in age between certain fracture types, while no significant differences are observed in BMI across the fracture types. Table 2 presents the results for age and BMI across different types of proximal femur fractures (medial, lateral, pertrochanteric, and subtrochanteric).

**Table 2:** Presents the mean and standard deviation (SD) of Age and Body Mass Index (BMI) across different types of proximal femur fractures (Medial, Lateral, Pertrochanteric, and Subtrochanteric). Significant differences between fracture types are indicated by p-values, with pairwise comparisons provided. Abbreviations: SD (Standard Deviation), Statistical analysis was conducted using ANOVA and post hoc pairwise comparisons. P-values less than 0.05 indicate significant differences between groups.

	medial	lateral	pertrochantaric	subtrochantaric			
	mean ± SD	mean ± SD	mean ± SD	mean ± SD	*p*-value	Lateral vs. pertrocharic	pertrochantaric vs. subtrochantaric
**Age**	75,07 ± 23,99	71,05 ± 24.35	79,52 ± 23,52	72.15 ± 25.40	<0.001	<0.001	0.002
**BMI**	12,49 ± 4.14	16,77 ± 4.74	12,58 ± 4.29	15.40 ± 5.02	0.065		

### 3.3 Correlations between anatomical measurements of the femur and body height

The Spearman correlation analysis reveals significant relationships between various anatomical measurements of the femur and body height (Table 3). Notably, there is a strong positive correlation between the FHA and both the horizontal (Spearman-Rho = 0.90, *p* < 0.001) and vertical femur head diameter (Spearman-Rho = 0.84, *p* < 0.001). Additionally, moderate to strong correlations were observed between the femur neck angle and body height (Spearman-Rho = 0.70, *p* < 0.001), and the femoral canal width 10 cm below the lesser trochanter (Spearman-Rho = 0.54, *p* < 0.001).

**Table 3:** The estimate is based on Fisher's r-to-z transformation. The estimate of the standard error is calculated using the formula proposed by Fieller, Hartley, and Pearson. Abbreviations: FHA = Femoral Head Area, FNA = Femoral Neck Axis Length (mm), FHD = Femoral Head Diameter, Canal width = Width of the medullary canal 10 cm below the lesser trochanter.

	Spearman-Rho	95 % CI		*p*-value
		lower bound	upper bound	
FHA - FNA	0.75	0.70	0.79	<0.001
FHA - FHD (horizontal)	0.90	0.88	0.92	<0.001
FHA - FHD (vertical)	0.84	0.80	0.86	<0.001
FHA- Canal width 10 cm below the lesser trochanter	0.54	0.46	0.61	<0.001
FHA - Body height	0.70	0.64	0.76	<0.001
FNA - FHD (horizontal)	0.74	0.70	0.78	<0.001
FNA - FHD (vertical)	0.64	0.58	0.70	<0.001
FNA- Canal width 10 cm below the lesser trochanter	0.51	0.43	0.58	<0.001
FNA - Body height	0.61	0.53	0.68	<0.001
FHD (horizontal) - FHD (vertical)	0.73	0.68	0.78	<0.001
FHD (horizontal) - Canal width 10 cm below the lesser trochanter	0.57	0.49	0.63	<0.001
FHD (horizontal) - body height	0.71	0.65	0.77	<0.001
FHD (vertical) - Canal width 10 cm below the lesser trochanter	0.48	0.39	0.55	<0.001
FHD (vertical) - body height	0.58	0.49	0.65	<0.001
Canal width 10 cm below the lesser trochanter - body height	0.34	0.24	0.44	<0.001

Further significant correlations include the femur neck diameter with the horizontal (Spearman-Rho = 0.74, *p* < 0.001) and vertical femur head diameter (Spearman-Rho = 0.64, *p* < 0.001), and with body height (Spearman-Rho = 0.61, *p* < 0.001). The horizontal and vertical femur head diameters themselves are also strongly correlated (Spearman-Rho = 0.73, *p* < 0.001).

These results highlight the strong associations between the femur neck angle, femur head diameters, and body height, supporting the hypothesis that certain anatomical measurements are interrelated.

### 3.4 Lateral and subtrochanteric fractures show significant differences in femoral head dimensions

**Fig. 4:**
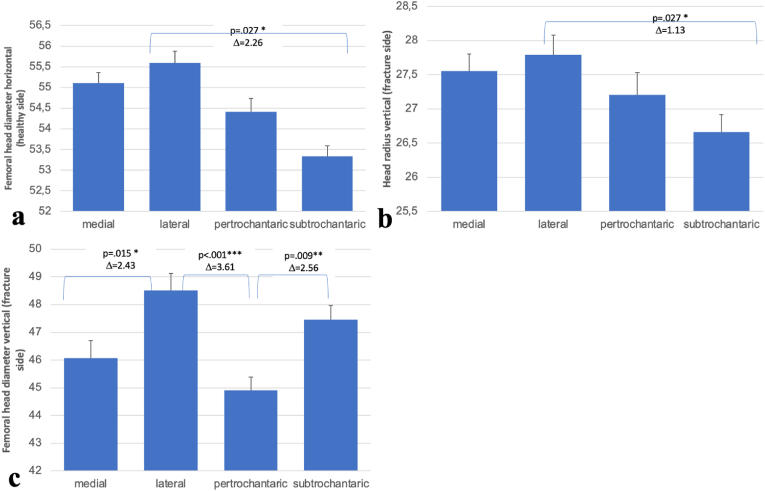
Morphological comparison of femoral head parameters across fracture types. **(a)** Femoral head diameter (horizontal, healthy side), **(b)** Head radius (vertical, fracture side), **(c)** Femoral head diameter (vertical, fracture side). Significant differences are indicated with p-values and Δ (mean difference) between the groups.

Fig. 4 shows graphical representations of the femoral head diameter (healthy side, horizontal), head radius (fracture side, vertical), and femoral head diameter (fracture side, vertical) in different types of proximal femur fractures. Significant differences are indicated with *p*-values and Δ (mean difference) between the groups.

In the first two graphs, it is noteworthy that lateral and subtrochanteric fractures show significant differences. In the first graph, depicting the horizontal femoral head diameter on the healthy side, there is a significant difference between lateral and subtrochanteric fractures (*p* = 0.027, Δ = 2.26). In the second graph, showing the vertical head radius on the fracture side, there is also a significant difference between lateral and subtrochanteric fractures (*p* = 0.027, Δ = 1.13).

However, the third graph, which represents the vertical femoral head diameter on the fracture side, shows no significant differences between lateral and subtrochanteric fractures. This could suggest that the fracture location has different impacts on the various measurements of the femoral head.

### 3.5 Comparison of femoral head parameters across fracture types

Table 4 provides a comparison of various femoral head parameters across different regions: medial, lateral, pertrochanteric, and subtrochanteric.

**Table 4:** Comparison of Femoral Head Area (FHA, fracture side), Centrum-Collum-Diaphyseal angle (CCD, healthy side), femoral head diameter horizontal (FHD, healthy side), femoral head length/height from midpoint (alternative to femoral neck shape), calculation of femoral neck shape (length of radius to neck axis length), femoral head diameter horizontal (FHD, fracture side), and femoral head diameter vertical (FHD, fracture side) across different regions: medial, lateral, pertrochanteric, and subtrochanteric. Values are presented as mean ± standard deviation (SD). The p-values indicate the statistical significance of differences between regions.

	medial	lateral	pertrochantaric	subtrochantaric	*p*-value
	mean ± SD	mean ± SD	mean ± SD	M	
**FHA (fracture side)**	2018.71 ± 394.24	2355,95 ± 2110.35	1972,96 ± 340.14	1995,72 ± 409.26	0.047
**CCD (healthy side)**	133.65 ± 5.59	133,3142 ± 5.80	133,27 ± 6.08	132,72 ± 6.33	0.740
**FHD horizontal (healthy side)**	55.11 ± 4.98	55.59 ± 5.75	54,41 ± 6.50	53,33 ± 5.07	0.028
**HRV (fracture side)**	27.56 ± 2.49	27.80 ± 2.87	27,21 ± 3.25	26,66 ± 2.54	0.028
**HRV/FNA**	0,25 ± 0.02	0,25 ± 0.02	0,25 ± 0.03	0,24 ± 0.02	0.384
**FHD horizontal (fracture side)**	54.68 ± 5.44	55.23 ± 6.35	53,93 ± 4.87	58,18 ± 52.63	0.693
**FHD vertical (fracture side)**	46.08 ± 6.28	48.51 ± 6.14	44,91 ± 4.81	47,46	<0.001

For the FHA on the fracture side, the *p*-value was 0.047, indicating a significant difference between the different groups. The largest FHA (*p* = 0.047) was found in the pertrochanteric group, as well as the largest horizontal diameter of the femoral head on the healthy side (*p* = 0.028) and head radius on the fracture side (*p* = 0.028) and the vertical diameter of the femoral head on the fracture side (*p* < 0.001).

## 4 Discussion

Our study provides significant insights into the influence of femoral head size and other factors on the localization of proximal femur fractures. The Spearman correlation analysis shows significant correlations between femur measurements and body height. Strong correlations are found between the FHA and both horizontal and vertical FHD. Femoral neck angle and diameter both showed significant correlations with body height and femoral head diameters, confirming that specific anatomical measurements are closely interrelated.

Patients with the youngest average age of 71 years had the largest FHAs and significantly more lateral fractures, suggesting that femoral head size may influence fracture localization. Additionally, osteoarthritis severity was highest in subtrochanteric fractures, suggesting hip joint degeneration impacts fracture localization. Age differences were significant between fracture types, particularly between lateral and pertrochanteric fractures and between pertrochanteric and subtrochanteric fractures, confirming that, older patients were at a higher risk for pertrochanteric fractures. Significant morphological differences were found in the vertical diameter of the femoral head on the fracture side and in the calculation of femoral head shape.

### 4.1 Clinical relevance

With increasing incidences of proximal femoral fractures, it is essential to recognize potential risk factors. This allows for the development of new guidelines and algorithms that can aid in diagnosis, biomechanical understanding, and patient education. A larger femoral head size may influence the biomechanical stress distribution, potentially increasing the risk of lateral proximal femur fractures. Addressing the biomechanical factors during treatment planning and ensuring targeted support for high-risk areas could enhance overall patient outcomes. The high prevalence of osteoarthritis, especially in subtrochanteric fractures, underscores the influence of degenerative changes in the hip joint on fracture localization and the necessity to consider osteoarthritis in treatment planning. For older patients, who have a higher risk of pertrochanteric fractures, age-specific strategies are crucial. Additionally, recognizing significant morphological differences in the hip can improve clinical risk assessment and enable more personalized patient care. These factors highlight the importance of individualized prevention and treatment approaches for different fracture types.

The study by Rotem et al. also examines the influence of hip morphology on the localization of proximal femur fractures and arrives at similar conclusions.^29^ Specifically, Rotem et al. identified significant differences in the alpha angle and neck-shaft angle between fracture types, suggesting that certain aspects of hip morphology may predispose patients to specific fracture patterns. These results support our findings, where parameters such as femoral head size and neck angle were also associated with fracture localization.

Our study identified a significant prevalence of osteoarthritis among different fracture types, especially subtrochanteric fractures, aligning with Rotem et al.'s findings of higher Tönnis classifications for hip osteoarthritis in extracapsular fractures.

While osteoarthritis is a common and expected finding in geriatric populations, its clinical relevance in this context lies in the observed correlation with specific fracture types. The presence of advanced degenerative changes may influence load distribution and joint mechanics, potentially predisposing patients to subtrochanteric fractures. This association highlights the importance of considering osteoarthritis not merely as a coincidental comorbidity, but as a biomechanical factor in fracture risk assessment and surgical decision-making.

Additionally, Tokyay et al. discuss the impact of acetabular morphology on the development of various proximal femur fracture types.^30^ They found that a higher acetabular index (AI) is associated with an increased risk of trochanteric fractures, while a greater hip axis length (HAL) is linked to an increased risk of femoral neck fractures. These findings complement those of Rotem et al., indicating that not only femoral head morphology but also acetabular morphology can play a role in determining the fracture type.^29^

Çukurlu et al. examined the effect of pre-fracture proximal femur geometry on hip fracture types in elderly patients and found similar trends.^31^ They reported that variations in proximal femur geometry significantly influence the type of hip fracture, aligning with our findings that femoral head size and shape are crucial determinants in fracture localization. Specifically, Çukurlu et al. found that an increased femoral head diameter (FHD) was associated with a higher risk of femoral neck fractures, which is consistent with our observation of the significant role of FHA in fracture localization. Furthermore, their findings that a higher neck shaft angle (NSA) and lower femoral offset (FO) values are linked to hip fractures support our results indicating the importance of these geometric parameters in fracture risk assessment.

The study by Patel et al. also supports our findings on the significance of proximal femur geometry.^32^ Patel et al. identified that the Hip Axis Length (HAL) and Femoral Neck Axis Length (FNAL) are significantly increased in extracapsular fractures compared to intracapsular fractures, especially in the older age group (61–90 years). This aligns with our observation that significant morphological differences, particularly in the vertical diameter of the femoral head and femoral head shape, play a crucial role in the localization of proximal femoral fractures. However, Patel et al. did not find significant associations for Femoral Head Diameter (FHD), Femoral Neck Diameter (FND), and Neck Shaft Angle (NSA), which contrasts with some of our findings where we observed morphological differences in these parameters.

The meta-analysis by Fajar et al. extends this discussion by showing that an increased hip axis length (HAL), a larger femoral neck angle (FNA), and a greater neck width (NW) are associated with a higher risk of femoral neck fractures.^33^ This underscores the importance of hip geometry in predicting fracture risks. The results of this meta-analysis are consistent with our own observations regarding the significance of morphological parameters. However, Fajar et al. found no significant association between femoral neck axis length (FNAL) and fracture risk, suggesting that certain geometric parameters may correlate differently with fracture risk.

Our study, while comprehensive, has some limitations that deserve consideration.

Although the study focused on femoral morphology, acetabular parameters – such as the acetabular index or bony coverage – were not included in the analysis. These aspects are known to influence hip biomechanics and fracture patterns and might have enhanced the interpretability of our results. Their exclusion represents a limitation and should be considered when interpreting the findings. This also highlights a valuable area for future research, in which a combined assessment of femoral and acetabular morphology may offer a more complete understanding of hip fracture mechanisms. First, being a retrospective analysis, there is a risk of biases due to missing or incomplete data. Additionally, the study was conducted at a single institution, which may limit the generalizability of the findings. It should be noted that the consecutive inclusion of 100 fractures of each type may have introduced a selection bias, potentially limiting the generalizability of the study results. A particularly intriguing aspect that warrants further exploration is the potential impact of projection phenomena related to post-fracture rotation on the measurements. These phenomena could vary depending on the type of fracture and might have influenced our results.

Through our study, several important clinical insights were gained. A larger femoral head size is associated with a higher risk of lateral proximal femur fractures, which is crucial for risk assessment and diagnosis. The high prevalence of osteoarthritis, especially in subtrochanteric fractures, demonstrates the influence of degenerative changes in the hip joint on fracture localization and underscores the need to include osteoarthritis in treatment planning. Older patients, with an average age of 79 years, have a higher risk for pertrochanteric fracture types. The study found that specific anatomical measurements of the femur, particularly the femur neck angle and femur neck diameter, are strongly correlated with both femur head diameters and body height. The study found no significant differences in the prevalence of other health conditions between fracture types, indicating that these comorbidities are less critical. These findings suggest that specific anatomical parameters significantly influence fracture localization.

## 5 Conclusion

Our findings indicate that a larger femoral head size is significantly associated with laterally localized proximal femur fractures, while older patients (mean age of 79 years) tend to experience pertrochanteric fractures. In contrast, subtrochanteric fractures predominantly occur in individuals with smaller femoral head dimensions and higher-grade osteoarthritis. These results underscore the pivotal role of specific anatomical features and degenerative changes in determining fracture localization and risk. Integrating femoral morphology and osteoarthritis severity into diagnostic and therapeutic approaches may enhance patient outcomes.

## CRediT authorship contribution statement

**Nele Wagener:** Conceptualization, Data curation, Formal analysis, Investigation, Methodology, Project administration, Software, Supervision, Validation, Visualization, Writing – original draft, Writing – review & editing. **Gregor Giebel:** Formal analysis, Software, Validation, Writing – review & editing. **Felix Rarreck:** Investigation, Software, Visualization, Writing – review & editing. **Torsten Diekhoff:** Data curation, Formal analysis, Funding acquisition, Investigation, Methodology, Software, Supervision, Validation, Visualization, Writing – review & editing. **Sebastian Hardt:** Conceptualization, Data curation, Formal analysis, Funding acquisition, Investigation, Methodology, Project administration, Resources, Software, Supervision, Validation, Writing – review & editing.

## Consent to participate

Informed consent was obtained from all individuals included in the study.

## Guardian/patient's consent

Patient consent was obtained. All analyses were performed in compliance with relevant laws and institutional guidelines and have been approved by the appropriate institutional committee(s).

## Ethics approval

The study was conducted in accordance with the Declaration of Helsinki, and received approval from the Institutional Review Board of Charité Universitätsmedizin Berlin, with the ethical consent number (EA1/203/23).

## Consent for publication

The authors affirm that human research participants provided informed consent for publication of the images Fig. 2, Fig. 3.

## Availability of data and material

The datasets used during the current study are available from the corresponding author on reasonable request.

## Ethics approval

The study was conducted in accordance with the Declaration of Helsinki, and received approval from the Institutional Review Board of Charité Universitätsmedizin Berlin, with the ethical consent number (EA1/203/23).

## Funding

The authors declare that no funds, grants, or other support were received during the preparation.

This research did not receive any specific grant from funding or agencies in the public, commercial or not-for-profit sectors.

## Declaration of competing interest

The authors declare that they have no known competing financial interests or personal relationships that could have appeared to influence the work reported in this paper.
